# Comparative Antioxidant, Anti-Acetylcholinesterase and Anti-α-Glucosidase Activities of Mediterranean *Salvia* Species

**DOI:** 10.3390/plants11050625

**Published:** 2022-02-25

**Authors:** Mateja Mervić, Maja Bival Štefan, Marija Kindl, Biljana Blažeković, Marijan Marijan, Sanda Vladimir-Knežević

**Affiliations:** Department of Pharmacognosy, Faculty of Pharmacy and Biochemistry, University of Zagreb, Trg Marka Marulića 20, 10000 Zagreb, Croatia; mmervic@pharma.hr (M.M.); mbival@pharma.hr (M.B.Š.); mkindl@pharma.hr (M.K.); bblazekovic@pharma.hr (B.B.); mmarijan@pharma.hr (M.M.)

**Keywords:** *Salvia* species, polyphenols, rosmarinic acid, antioxidant activity, acetylcholinesterase inhibition, α-glucosidase inhibition

## Abstract

*Salvia* species have a cosmopolitan distribution and comprise several well-known plants valuable for pharmaceutical and food industries due to their recognized medicinal, food flavouring, and preservative properties. The present study aimed to evaluate and compare the biological activities of seven wild-growing *Salvia* species from the Mediterranean area (*S. fruticosa*, *S. glutinosa*, *S. nemorosa*, *S. officinalis*, *S. pratensis*, *S. sclarea*, *S. verticillata*). All studied ethanolic leaf extracts exhibited significant DPPH and NO radical scavenging ability, lipid peroxidation inhibition, and reducing power, as well as moderate iron-chelating properties. Together with *S. officinalis* and *S. fruticosa*, *S. verticillata* showed anti-acetylcholinesterase activity, while *S. glutinosa* was also found to possess the ability to inhibit α-glucosidase. Total flavonoid (0.37–0.90%), phenolic acid (3.55–12.44%), tannin (1.22–2.60%), and anthocyanin contents (0.03–0.08%) were determined in *Salvia* leaves. Rosmarinic acid was the predominant hydroxycinnamic acid in all studied sage plants, ranging from 9400 to 38,800 μg/g. The correlation study showed a strong relationship between biological activities and contents of total phenolic acids, total tannins, and rosmarinic acid, indicating their significant contribution to the efficiency of tested *Salvia* species. Our results highlighted Mediterranean sage plants as rich sources of potent antioxidant, neuroprotective, and hypoglycemic agents which are worthy of further research.

## 1. Introduction

*Salvia* L. is the largest genus of the Lamiaceae family. It comprises approximately 1000 species and has a cosmopolitan distribution. The main speciation centres of these taxa are Central and South America, Central Asia and the Mediterranean region, and East Asia. The genus *Salvia* includes annual, biennial, and perennial herbs, and woody subshrubs that exhibit remarkable diversity in growth forms, floral morphology, pollination biology and secondary metabolites [1,2]. *Salvia* species, commonly known as sage, are useful plants which have not lost their importance since ancient times. Some of them, such as *S. officinalis* L. (Dalmatian sage, common sage), *S. lavandulifolia* Vahl (Spanish sage), *S. sclarea* L. (clary sage), *S. fruticosa* Mill. (Greek sage), *S. miltiorrhiza* Bunge (danshen) and *S. hispanica* L. (chia), are cultivated worldwide for use in the pharmaceutical and food industries [3,4]. The main secondary metabolites of *Salvia* species are terpenoids and polyphenols. They are a rich source of essential oils with large contents of α- and β-thujone, camphor, 1,8-cineole, α-humulene, β-caryophyllene, and viridiflorol. Diterpenes and triterpenes, such as carnosol, carnosic acid, tanshinones, and ursolic acid, are also very abundant compounds. The most representative flavonoids in the sage plants are the flavones apigenin and luteolin, their corresponding 6-hydroxylated derivatives, and flavonols. Caffeic acid present in various *Salvia* species is the building block of several metabolites from simple monomers to oligomers. The most abundant caffeic acid dimer identified in sage plants is rosmarinic acid, while lithospermic and salvianolic acids, sagecoumarin and yunnaneic acids are predominant among trimers derived from caffeic acid [5,6,7]. Previous research revealed the extensive biological effects of *Salvia* species, including antimicrobial, antioxidant, anti-inflammatory, neuroprotective, and anticancer activities, among others [7,8,9,10]. 

The plants of the genus *Salvia* are widespread in tropical and temperate regions of Europe around the Mediterranean area to which Croatia belongs [8]. Croatian flora comprises 19 *Salvia* taxa, one of which is endemic [11]. The important representative of the genus *Salvia* in Croatian flora is *S. officinalis* L. It is an aromatic perennial subshrub, with woody stems, greenish-grey leaves, and rosy to violet-blue flowers, native to the Mediterranean shores [12,13]. Dalmatian sage is one of the most commercially important species within the mint family known for its richness in essential oils and its wide range of healing properties. The sage leaf (Salviae officinalis folium) is officially recognized as the herbal drug for the relief of inflammations in the mouth or the throat, mild dyspeptic complaints, excessive sweating, and minor skin inflammations [14]. In addition, *S. officinalis* has been used for a long time in food preparation due to its spicy, appetizing, digestive, and food preservative properties. It is considered as GRAS (Generally Recognized as Safe) for use as a spice and other natural seasonings and flavourings based on the Food and Drug Administration [15,16]. 

Essential oils, non-volatile terpenes, flavonoids, and phenolic acids are proven to be the main active principles of *S. officinalis* that greatly contribute to its pharmacological properties [6,16,17]. The composition of essential oils can vary widely, but the major components are mostly oxygenated monoterpenoids α-thujone, 1,8-cineole, and camphor [12,18]. Diterpenes and triterpenes (carnosic acid, carnosol, and ursolic acid) are also found to be present in Dalmatian sage [19,20]. The most prevalent flavonoids include glycosides of luteolin, apigenin, hispidulin, and quercetin [21,22,23]. Rosmarinic, salvianolic, caffeic and sagerinic acids, and sagecoumarin are abundant phenolic acids of *S. officinalis* [23]. In recent years, many studies have been conducted to prove traditionally claimed applications of *S. officinalis* and to discover new therapeutic properties of this plant. These studies revealed a wide range of pharmacological activities including antioxidant, anti-inflammatory, antimicrobial, anticancer, antimutagenic, hypoglycemic, antinociceptive, memory-enhancing, and hypolipidemic effects. Moreover, the last three listed have been demonstrated with clinical trials [16]. 

Along with *S. officinalis*, the important representatives of the genus *Salvia* wild growing in Croatia are *S. fruticosa* Mill., *S. glutinosa* L., *S. nemorosa* L., *S. pratensis* L., *S. sclarea* L., and *S. verticillata* L., which have not yet been sufficiently investigated. Due to the lack of scientific evidence, the present study aimed to evaluate the antioxidant, neuroprotective, and antidiabetic potential of the ethanolic extracts of selected *Salvia* species in comparison with *S. officinalis* as the most studied among the sage plants. The research focused specifically on plant polyphenolic constituents that exhibit strong antioxidant properties and have the potential to be used as dietary and therapeutic agents and food preservatives.

## 2. Materials and Methods

### 2.1. Chemicals

HPLC grade acetonitrile and o-phosphoric acid were provided by VWR Chemicals (Fontenay-sous-Bois, France). Acetylcholinesterase from electric eels and acetylthiocholine iodide (>98%), α-glucosidase from *Saccharomyces cerevisiae* (Type I, >10 U/mg protein), 2-amino-2-(hydroxymethyl)propane-1,3-diol (Tris base), *p*-anisaldehyde, brain extract from bovine brain, chlorogenic acid (95%), *p*-coumaric acid (98%), 2,2-diphenyl-1-picrylhydrazyl (DPPH), 5,5′-dithiobis-2-nitrobenzoic acid (DTNB, >98%), hide powder, phosphate buffer saline, 4-nitrophenyl-α-D-glucopyranoside (>99%), pyrogallol (99%), rosmarinic acid (97%), sodium molybdate, sodium nitrite, sulphanilamide, thiobarbituric acid, and trichloroacetic acid were purchased from Sigma-Aldrich (St. Louis, MO, USA). Trolox (>98%), 3-(2-pyridyl)-5,6-diphenyl-1,2,4-triazine-4′,4′′-disulfonic acid sodium salt (ferrozine), butylhydroxytoluene (BHT), caffeic acid (>95%), ferulic acid (>98%), and N-(1-naphthyl)ethylenediamine dihydrochloride were provided by Fluka (Buchs, Switzerland). Acarbose (>98%) and α/β-thujone were provided by TCI (Tokyo, Japan). Aluminum chloride hexahydrate, disodium hydrogen phosphate dihydrate, ethylenediaminetetraacetic acid disodium salt (EDTA), formic acid, hexamethylenetetramine, iron(II) sulphate heptahydrate, sodium dihydrogen phosphate dihydrate, and sodium sulphate were obtained from Kemika (Zagreb, Croatia). Acetone, dimethyl sulfoxide, ethanol, and sodium carbonate decahydrate were purchased from Gram-Mol (Zagreb, Croatia). Acetic acid and sodium hydroxide were provided by Carlo Erba (Rodano, Italy). Ascorbic acid and phosphomolybdic acid were obtained from Acros Organics (Geel, Belgium). Iron(III) chloride was purchased from Riedel-de-Haën (Seelze, Germany). Folin-Ciocalteau’s phenol reagent, potassium hexacyanoferrate(III), and sodium nitroprusside were purchased from Merck (Darmstadt, Germany). Methanol, phosphoric acid, and toluene were provided by T.T.T. (Zagreb, Croatia). Butanol, hydrochloric acid, and methanol (HPLC grade) were obtained from Lach-Ner (Neratovice, Czech Republic). Ethyl acetate, sulphuric acid, and hydrochloric acid were purchased from POCH S.A. (Gliwice, Poland).

### 2.2. Plant Material and Extract Preparation

The leaves of seven selected *Salvia* species were collected during the flowering period between May and August 2018 from different locations in Croatia as follows: *S. fruticosa* Mill. (Vis, 43°03′01.3′′ N, 16°04′14.5′′ E), *S. glutinosa* L. (Čabar, 45°27′42.6′′ N, 14°49′36.4′′ E), *S. nemorosa* L. (Baranja, 45°49′34.1′′ N, 18°45′29.3′′ E), *S. officinalis* L. (Dugi otok, 44°04′47.0′′ N, 14°59′40.7′′ E), *S. pratensis* L. (Zagreb, 45°47′04.6′′ N, 15°59′51.3′′ E), *S. sclarea* L. (Dugi otok, 44°04′47.0′′ N, 14°59′40.7′′ E), and *S. verticillata* L. (Ogulin, 45°18′32.7′′ N, 15°16′44.5′′ E). The plant species were authenticated by the Department of Botany, Faculty of Science and the Department of Pharmacognosy, Faculty of Pharmacy and Biochemistry (University of Zagreb, Croatia), where the voucher specimens were deposited under the genus number 817. In order to investigate the biological activities of selected *Salvia* species, air-dried plant material was pulverized into a fine powder and used for extract preparation. Afterward, 50 mL of 70% ethanol was added to 5.0 g of pulverized plant material and the extraction procedure was carried out in an ultrasonic bath (Bandelin Sonorex digital 10P, Germany) for 30 min at 30 °C. After filtration, the sample was reextracted after which a new portion of 50 mL of 70% ethanol was added to the plant residue. The newly obtained extract was filtered after the extraction procedure was repeated in the above-mentioned conditions. The filtrates were combined and concentrated to dryness with a rotary evaporator (Büchi Rotavapor, Switzerland). The extraction yields for *S. fruticosa*, *S. glutinosa*, *S. nemorosa*, *S. officinalis*, *S. pratensis*, *S. sclarea,* and *S. verticillata* were 26.17, 18.00, 23.56, 34.17, 30.80, 22.19, and 34.39%, respectively. Additionally, *Salvia* ethanolic extracts were analysed for thujone presence by thin-layer chromatography according to method A described in the European Pharmacopoeia [24], as well as method B by Wagner and Bladt [25]. The mobile phase containing ethyl acetate and toluene in proportions 5:95 *v/v* (A) or 7:93 *v/v* (B) was used. Aliquots of 0.1% α/β-thujone (20 μL) and 1% *Salvia* extracts (10 μL) were manually applied on a silica gel HPTLC plate (Merck, Germany). The plate was sprayed with a solution of phosphomolybdic acid in 96% ethanol (A) or anisaldehyde-sulphuric acid reagent (B) and heated at 100–105 °C for 10 min. Neither *Salvia* chromatograms examined in daylight showed any pale blue (A) or violet zones (B) matching R_f_ value as that of thujone in the reference solution. 

### 2.3. Total Flavonoids Determination

The total flavonoid contents in the leaves of selected *Salvia* species were determined spectrophotometrically according to the method described in the European Pharmacopoeia [24]. Firstly, 0.600 g of pulverized plant material was extracted with 1.0 mL of hexamethylenetetramine (5 g/L), 20 mL of acetone, and 2.0 mL of hydrochloric acid (250 g/L) in a water bath under reflux for 30 min. The mixture was cooled and filtered through cotton and the residue was extracted twice with 20 mL of acetone for 10 min. The filtrates were combined and diluted with acetone to 100.0 mL. Afterward, 20.0 mL of the obtained acetone extract was mixed with 20 mL of distilled water and extracted once with 15 mL and three times with 10 mL of ethyl acetate. The ethyl acetate fractions were combined and washed twice with 50 mL of distilled water, filtered through anhydrous sodium sulphate, and diluted with ethyl acetate to 50.0 mL. Afterward, 10.0 mL of the ethyl acetate extract was mixed with 1.0 mL of an aluminium chloride solution and diluted with a 5% methanolic solution of acetic acid to 25.0 mL. The absorbance of the test solution was measured after 30 min at 425 nm, after which the percentage of total flavonoids, expressed as isoquercitrin, was calculated according to the following equation: (%) = *A* × 1.25/*m*. *A* represents the absorbance of the test solution whereas *m* represents the mass of used plant material in grams. 

### 2.4. Total Phenolic Acids Determination

The total phenolic acid contents in the leaves of selected *Salvia* species were determined spectrophotometrically according to the European Pharmacopoeia [24]. Firstly, 0.200 g of pulverised leaves were extracted with 50% ethanol in a water bath under reflux. After cooling and filtration, the extract was diluted to 100.0 mL with 50% ethanol. Afterward, 1.0 mL of the extract was mixed with 2.0 mL of 0.5 M hydrochloric acid, 2.0 mL water solution of sodium nitrate and sodium molybdate, as well as 2.0 mL of 8.5% sodium hydroxide and diluted using distilled water to 10.0 mL. The absorbance of the test solution was measured immediately at 505 nm and the percentage of total phenolic acids, expressed as rosmarinic acid, was calculated according to the following expression: (%) = *A* × 2.5/*m*. *A* stands for the absorbance of the test solution while *m* stands for the mass of tested plant material in grams.

### 2.5. Total Tannins Determination

The total tannin contents in the leaves of selected *Salvia* species were determined spectrophotometrically using the assay described in the European Pharmacopoeia [24]. Firstly, 1.000 g of pulverized leaves were extracted with distilled water in a water bath under reflux. After cooling, the water extract was filtered, transferred into a volumetric flask, diluted to 250.0 mL, and filtered. After discarding the first 50 mL, 10.0 mL of the filtrate was mechanically stirred with the addition of hide powder for 60 min, filtrated, and diluted with water to 25.0 mL, after which 2.0 mL of said solution was mixed with 1.0 mL of the Folin-Ciocalteau’s reagent, as well as 10.0 mL of water. The mixture was diluted to 25.0 mL with sodium carbonate (290 g/L) and the absorbance was measured after a 30-min incubation at 760 nm. The content of total tannins, expressed as pyrogallol, was calculated according to the following expression: (%) = 62.5 × (*A*_1_ − *A*_2_) × *m*_2_/*A*_3_ × *m*_1_. *A*_1_ stands for the absorbance of the test solution, which was not pre-treated with hide powder; *A_2_* represents the absorbance of the test solution treated with hide powder; *A*_3_ stands for the absorbance of the standard pyrogallol; *m*_1_ represents the mass of tested plant material in grams, and *m*_2_ stands for the mass of pyrogallol.

### 2.6. Total Anthocyanins Determination

The total anthocyanin contents in the leaves of selected *Salvia* species were determined spectrophotometrically following the assay described in the European Pharmacopoeia [24]. Firstly, 5.00 g of pulverized leaves were mixed with methanol and mechanically stirred for 30 min. The methanolic extract was filtered and diluted to 100.0 mL with methanol. The solution was once again diluted 50 times with a 0.1% methanolic solution of hydrochloric acid. The absorbance was measured immediately at 528 nm. The content of total anthocyanins, expressed as cyanidine-3-glucoside chloride, was calculated using the following equation: (%) = *A* × 5000/718 × *m*. *A* represents the absorbance of the test solution whereas *m* represents the mass of tested plant material in grams.

### 2.7. Determination of Phenolic Acids and Flavonoids by High-Performance Liquid Chromatography

The contents of phenolic acids in the leaves of selected *Salvia* species were determined by the high-performance liquid chromatography method described in the European Pharmacopoeia [24]. The analysis was performed using the Agilent 1100 HPLC system with Zorbax RRHD Eclipse plus C18 column (4.6 × 250 mm, 5 µm). Firstly, pulverised leaf material (0.100 g) was extracted for 30 min with 90 mL of 50% ethanol in a water bath under reflux. The extract was cooled and filtered into a volumetric flask, diluted to 100.0 mL with 50% ethanol, then additionally filtered through a 0.45 μm syringe filter. The reference solutions of the phenolic acids were prepared by dissolving 20.0 mg of each in 50% ethanol and diluted to 100.0 mL. The solution was diluted fivefold and filtered through a 0.45 μm filter. Mobile phase A consisted of phosphoric acid, acetonitrile, and water (1:19:80 *v/v/v*), while mobile phase B contained phosphoric acid, methanol, and acetonitrile (1:40:59 *v/v/v*). Eluents were used in the following gradient program: 0–20 min (0–45% B), 20–25 min (45–100% B), and 25–30 min (100–0% B). The flow rate was adjusted to 1.2 mL/min. Phenolic acid detection was conducted at 330 nm. The amounts of rosmarinic, chlorogenic, *p*-coumaric, caffeic, and ferulic acids were calculated by using the peak area normalisation procedure and the results were expressed as μg of active substance per g of herbal material.

The quantification of apigenin, apigenin-7-O-glucoside, luteolin, luteolin-7-O-glucoside, quercetin, and rutin in the *Salvia* extracts was carried out according to the method previously described by Bljajić et al. [26]. The analysis was performed using the Agilent HPLC system (Agilent 1200 series, Agilent Technologies, Santa Clara, CA, USA) with the Eclipse XDB-C18 column (4.6 × 250 mm, 5 µm) and guard column, equipped with an autosampler as well as a DAD detector. Firstly, 1.0 g of corresponding herbal material was extracted for 15 min with 10 mL of 96% ethanol in a water bath under reflux. The extract was cooled and then filtered through a 0.45 μm syringe filter. The phenolic standards (0.1–0.2 mg/mL) were also filtered and subjected to HPLC chromatographic separation. Mobile phase A (water, methanol, and formic acid in proportions 93:5:2 *v/v/v*) and mobile phase B (water, methanol, and formic acid in proportions 3:95:2 *v/v/v*) were utilized in the following order: 0 min 20% B, 10 min 40% B, and 35 min 50% B. The separation was performed at 40 °C and at the flow of 1.0 mL/min. All flavonoids were detected and quantified at 270 nm using corresponding calibration curves. The peak assignment and identification were based on the comparison of UV/VIS spectra and retention times of the peaks in the sample chromatograms with those of the standards. The results were expressed as μg of active substance per g of herbal material. 

### 2.8. DPPH Radical Scavenging Activity

The DPPH radical scavenging activity of the ethanolic extracts of selected *Salvia* species was determined according to the method described by Vladimir-Knežević et al. [27]. Briefly, the solutions of plant extracts and the reference standards in different concentrations (0.78–50 µg/mL) were prepared in ethanol, respectively. Afterward, a 0.1 mM solution of DPPH was prepared in ethanol as well, and 0.5 mL of said reagent was then added to 1.5 mL of test solutions. After being vigorously shaken, the samples were kept in the dark for 30 min. Their absorbance was measured afterward at 517 nm against an appropriate blank. The DPPH radical scavenging capability was calculated according to the following equation: (%) = [(*A*_0_ − *A*_1_)/*A*_0_] × 100. *A*_0_ represents the absorbance of the control solution whereas *A*_1_ represents the absorbance of the test solution.

### 2.9. NO Radical Scavenging Activity

The NO radical scavenging activity of selected *Salvia* species was tested according to the method used by Patel et al. [28] with slight modifications. Ethanolic plant extracts and the reference standards were prepared in different concentrations (6.25–400 µg/mL). Afterward, 1.0 mL of freshly prepared 10 mM solution of sodium nitroprusside in phosphate-buffered saline (pH = 7.4) was added to 1 mL of test solutions. The test tube contents were shaken vigorously and incubated at room temperature for two hours. Afterward, 0.5 mL of tested samples were transferred in a new set of test tubes and mixed with 1.0 mL of 1% solution of sulphanilamide in 5% phosphoric acid. After a 5-min incubation at room temperature, 1.0 mL of 0.1% solution of N-(1-naphthyl)ethylenediamine dihydrochloride was added to the samples and their absorbance was measured immediately after shaking at 540 nm with the use of an appropriate blank. NO radical scavenging activity was calculated with the following equation: (%) = [(*A*_0_ − *A*_1_)/*A*_0_] × 100, where *A_0_* is the absorbance of the control solution and *A*_1_ is the absorbance of the test solution.

### 2.10. Reducing Power Assay

The reducing power of seven *Salvia* species was determined according to the assay described by Vladimir-Knežević et al. [27]. A series of ethanolic extract solutions and reference standards were prepared in different concentrations (1.56–100 µg/mL). After the addition of 2.5 mL of 0.2 M phosphate buffer (pH = 6.6) and 2.5 mL of 1% solution of potassium hexacyanoferrate(III), the samples were incubated for 20 min at 50 °C. Then, 2.5 mL of 10% solution of trichloroacetic acid was added to the test solutions. Afterwards, 2.5 mL of the supernatant was mixed with 2.5 mL of distilled water and 0.5 mL of 0.1% solution of iron(III) chloride, and the absorbance of tested samples was measured at 700 nm against a blank.

### 2.11. Iron Chelating Activity

The iron-chelating activity of *Salvia* species in comparison with EDTA as the reference chelator was determined according to the slightly modified method conducted by Benabdallah et al. [29]. Firstly, ethanolic solutions of plant extracts and a water solution of EDTA were prepared in different concentrations (100–1600 µg/mL and 0.78–12.5 µg/mL, respectively). After adding 0.3 mL of 0.1 mM solution of iron (II) sulphate heptahydrate to 0.3 mL of test solutions, the mixture was incubated for 10 min at room temperature. After the addition of 0.3 mL of 0.25 mM solution of ferrozine in the next step, the vigorously shaken samples were once again incubated at room temperature for the same amount of time and their absorbance was promptly measured at 562 nm with the use of a blank. The percentage of iron-chelating ability was calculated according to the following equation: (%) = [(*A*_0_ − *A*_1_)/*A*_0_] × 100. *A*_0_ represents the absorbance of the control solution while *A*_1_ represents the absorbance of the test solution.

### 2.12. Lipid Peroxidation Inhibition Assay

The lipid peroxidation inhibition assay for seven selected *Salvia* species was carried out according to the method described by Houghton et al. [30]. Ethanolic plant extracts and rosmarinic acid as the reference standard were prepared in dimethyl sulfoxide in different concentrations (50–400 µg/mL and 12.5–100 µg/mL, respectively). Afterward, 10 µL of each test solution was mixed with 0.5 mL of the bovine brain extract suspension in phosphate-buffered saline (5 mg/mL, pH = 7.4). After the addition of 0.1 mL of 1 mM solution of iron(III) chloride, 0.29 mL of 10 mM phosphate-buffered saline, and 0.1 mL of 1 mM solution of ascorbic acid, respectively, the test solutions were incubated for 60 min at 37 °C. After incubation, 1 mL of 1% solution of thiobarbituric acid in 0.05 M sodium hydroxide, 1 mL of 2.8% solution of trichloroacetic acid, and 0.1 mL of 2% solution of BHT were added into the mixture. The test solutions were then heated in a boiling water bath for 20 min. After cooling, 2 mL of butanol was added to the mixture after which 1 mL of the samples’ supernatant was transferred into new test tubes. The absorbance of tested samples was measured at 532 nm against an appropriate blank and the percentage of lipid peroxidation inhibition was calculated by using the following equation: (%) = [(*A*_0_ − *A*_1_)/*A*_0_] × 100. *A*_0_ stands for the absorbance of the control solution while *A*_1_ stands for the absorbance of the test solution.

### 2.13. Acetylcholinesterase Inhibition Assay

The acetylcholinesterase inhibition assay for tested *Salvia* species was conducted according to the method developed by Conforti et al. [31]. Solutions of plant extracts, rosmarinic acid, and galantamine were prepared in dimethyl sulfoxide in different concentrations (100–1600 µg/mL). Afterward, 20 µL of each test solution was mixed with 40 µL of an acetylcholinesterase solution (0.02 U/mL) and 1.9 mL of 50 mM Tris-HCl buffer (pH = 8), after which the samples were kept at 4 °C for 30 min. After incubation, 40 µL of a mixture of 10 mM solution of 5,5′-dithiobis-2-nitrobenzoic acid and 12 mM solution of acetylthiocholine iodide was added to the samples and their absorbance was measured promptly after stirring and once again after ten minutes at 412 nm. The percentage of acetylcholinesterase inhibition was obtained according to the following equation: (%) = [(*A*_0_ − *A*_1_)/*A*_0_] × 100. *A*_0_ stands for the difference in the absorbance of the control solution measured immediately and ten minutes later, whereas *A*_1_ stands for the difference in the absorbance of the test solution measured immediately and ten minutes later.

### 2.14. α-Glucosidase Inhibition Assay

The α-glucosidase inhibition assay for tested *Salvia* species was carried out according to the method described by Bljajić et al. [32] with slight modifications. Solutions of the plant extracts (100 μL) were prepared in 10% dimethyl sulfoxide in different concentrations (400–6400 μg/mL). Afterward, the solutions were mixed with 50 μL of the α-glucosidase solution (1.0 U/mL), which was prepared in 0.1 M phosphate buffer (pH = 6.8), and incubated for 10 min at 37 °C. After incubation, 50 μL of the substrate 4-nitrophenyl-α-glucopyranoside (1.25 mM) prepared in the same buffer was added to the mixture. Absorbance was measured at 405 nm after a 5-min incubation at room temperature against a blank which contained the equivalent volume of phosphate buffer instead of the substrate. The antidiabetic drug acarbose was used as the positive control during the experiment while a solution containing 10% dimethyl sulfoxide instead of plant extracts were used as the negative control. The enzyme inhibitory activity was calculated according to the following equation: (%) = [(*A*_0_ − *A*_1_)/*A*_0_] × 100. *A*_0_ stands for the absorbance of the control solution which contains 10% dimethyl sulfoxide instead of tested samples, whereas *A*_1_ represents the absorbance of the solution which contains either *Salvia* extracts or acarbose.

### 2.15. Statistical Analysis

All the above-mentioned experiments were carried out in triplicates and the results are expressed as mean ± standard deviation. The IC_50_ values were calculated by linear regression extrapolation. The differences between the obtained results were determined by the ordinary one-way ANOVA and post hoc Tukey’s multiple comparisons test. Pearson’s correlation coefficient was calculated to find the degree of association between the two variables. All statistical analyses were accomplished by the GraphPad Prism software (version 8.4.3) as well as Microsoft Excel. All values with *p* < 0.05 were considered statistically significant.

## 3. Results and Discussion

### 3.1. Phytochemical Analysis of Polyphenolic Compounds in Selected Salvia Species

Along with other species of the mint family, the sage plants are being recognized as a promising source of polyphenols that exhibit a remarkable diversity of both chemical structures and biological activities [2,8,33]. The contents of different groups of polyphenolic components, phenolic acids, flavonoids, tannins, and anthocyanins, in the leaves of selected *Salvia* species, were determined spectrophotometrically and obtained results are presented in Table 1. The most abundant compounds for all the investigated species were phenolic acids (3.55–12.44%), followed by tannins (1.22–2.60%), and flavonoids (0.31–1.07%). As expected, the percentage of anthocyanins was very low (0.02–0.08%). The leaves of *S. verticillata* contained the highest amount of phenolic acids (12.44%) while the lowest concentration was determined in *S. sclarea* (3.55%). The contents of phenolic acids in the other sage plants were from 6.48% to 8.04%. Among studied *Salvia* species, *S. glutinosa* was found to contain the largest amount of tannins (2.60%), flavonoids (1.07%), and anthocyanins (0.08%). In addition to *S. glutinosa*, *S. nemorosa*, *S. pratensis,* and *S. sclarea* were also rich in flavonoids (0.75–0.90%). 

Previous research on *Salvia* species frequently involved the spectrophotometric determination of total phenols, phenolic acids, and/or flavonoids in some of the tested sage plants with the aim to correlate their contents and bioactivity. Zupkó et al. [34] determined the total phenol and flavonoid contents in several *Salvia* species, including *S. glutinosa*, *S. nemorosa*, *S. officinalis*, *S. pratensis,* and *S. verticillata*. Except for *S. glutinosa* (2.5%), 50% of methanolic extracts contained less than 1% of flavonoids. On the other hand, the proportion of total phenols among tested *Salvia* extracts varied considerably (1–26%). Previous reports also indicate a great variability in the total phenolics (expressed as gallic acid equivalents) of *Salvia* extracts of various polarity as follows: *S. officinalis* (2–293 mg/g) [35,36,37,38], *S. fruticosa* (24–267 mg/g) [39,40,41,42,43], *S. nemorosa* (40–295 mg/g) [44,45,46], *S. sclarea* (30–268 mg/g) [41,42,44,47,48], and *S. verticillata* (50–176 mg/g) [46,47,49]. Contrary to the above, the total phenolics in different samples of *S. glutinosa* were quite similar (38–43 mg/g) [44,50]. Apart from *S. fruticosa* which contained flavonoids in a wide range from 4 to 346 mg rutin/g [43,51], the variability in the total flavonoids and phenolic acids within and between *Salvia* species was significantly less than that of total phenols. The content of total flavonoids in *S. glutinosa* was 16 mg rutin/g [50], while 2 mg rutin/g was determined in both *S. sclarea* and *S. verticillata* [47,49]. Furthermore, *S. nemorosa*, *S. sclarea,* and *S. verticillata* were found to contain 29, 26, and 39–42 mg/g of total phenolic acids expressed as caffeic acid equivalents, respectively [47]. Due to the differences in the type of extract and plant parts used, determination method, or expression of the results, it is difficult to accurately compare previous findings with each other and with our results. However, in accordance with previous research, our study clearly revealed that all investigated *Salvia* extracts can be considered as rich sources of polyphenols. Furthermore, we confirmed that our *Salvia* ethanolic extracts do not contain α/β-thujone, which contributes to the safety aspects of their potential application.

Since phenolic acids were found to be the major class of phenolic compounds for all studied *Salvia* species, the contents of selected phenolic acids were additionally determined by the HPLC-DAD method. Rosmarinic, chlorogenic, *p*-coumaric, caffeic, and ferulic acids were quantified due to their frequent occurrence in sage plants [39,49,52]. According to the results presented in Table 2, rosmarinic acid was the most abundant in the leaves of all investigated plants, which is consistent with previously reported data [35,39,53]. The contents of rosmarinic acid were ranging from 9400 μg/g (*S. glutinosa*) to 38,800 μg/g (*S. officinalis*). The highest percentage of chlorogenic and *p*-coumaric acid was also found in the leaves of *S. officinalis* (2100 and 11,600 μg/g, respectively). *S. fruticosa* and *S. verticillata* contained a significant amount of rosmarinic acid (29,100 and 30,200 μg/g, respectively) and were the richest in caffeic acids. Moreover, ferulic acid was detected only in the leaves of those two species. When comparing our findings with the previous phytochemical studies, both similarities and differences can be observed. The highest concentration of rosmarinic acid in *Salvia officinalis* was also reported by Farhat et al. [35]; however, our study highlighted the Croatian specimen as a much richer source than the Tunisian one (13,680–18,378 μg/g). Alongside this, the concentrations of rosmarinic acid in *S. officinalis* and *S. fruticosa* reported by Cvetkovikj et al. [53] were 25.98 mg/g and 10.72 mg/g, respectively, which is significantly lower compared to our results. According to Šulniūtė et al. [54], who studied ten *Salvia* spp. growing in Lithuania, the major phenolic acids were rosmarinic and caffeic acids. Their contents in the ethanolic extracts of *S. glutinosa* (9225 and 630 μg/g, respectively) and *S. officinalis* (30,017 and 605 μg/g, respectively) were in line with our results, despite a different extraction method. However, *S. verticillata* and *S. pratensis* analysed in our study contained the two- and threefold higher amounts of rosmarinic acid, respectively. Comparable to our findings, Katanić Stanković et al. [49] also reported higher rosmarinic acid content in Serbian *S. verticillata*.

As can be seen in Table 2, apigenin, luteolin, and their glycosidic derivatives were detected in the studied *Salvia* extracts, along with quercetin and rutin, which are all typical flavonoids present in sage plants [49,51,53]. However, the amounts of analysed flavonoids in tested samples were much lower than that of phenolic acids. The most common flavonoids found were luteolin and luteolin-7-O-glucoside. Their concentrations in *Salvia* leaves ranged from 11.86 μg/g (*S. fruticosa*) to 163.54 μg/g (*S. sclarea*) and from 51.73 μg/g (*S. glutinosa*) to 306.81 μg/g (*S. fruticosa*), respectively. Apigenin-7-O-glucoside was identified only in *S. glutinosa* (156.83 μg/g), although its aglycone apigenin was quantified in *S. sclarea*, *S. officinalis,* and *S. fruticosa*. Similarly, the extracts of *S. fruticosa* and *S. verticillata* contained quercetin, while its glycosidic derivative was detected only in *S. officinalis* and *S. pratensis*. Our study shows that *S. officinalis*, besides rutin, contains apigenin, luteolin, and their glucosides, but their concentrations were remarkably lower in comparison to a previous report [35]. Sarrou et al. [51] reported that the phenolic content and composition of *S. fruticosa* is greatly influenced by the harvest period, and found, contrary to our results, that luteolin is more present as the free aglycone. According to our findings, none of the monitored flavonoids have been detected in *S. nemorosa*, which is not in line with the previous papers [45,47] that reported quercetin and rutin. However, our results on the presence of luteolin and apigenin in *S. sclarea* support previous findings [47,52]. Hanganu et al. [47] quantified luteolin, apigenin, and rutin in the ethanolic extracts of *S. verticillata,* but did not detect quercetin, which indicates the great variations in the flavonoid composition of this sage species. 

### 3.2. Antioxidant Activities of Selected Salvia Species

**Figure 1:**
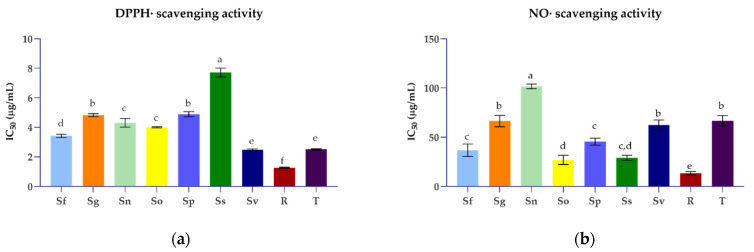

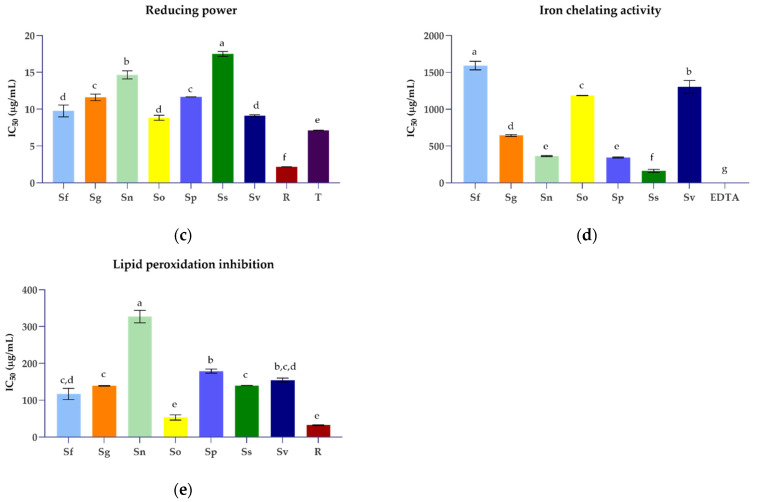
Comparative overview of antioxidant effects (IC_50_ values) of the ethanolic extracts of *S. fruticosa* (Sf), *S. glutinosa* (Sg), *S. nemorosa* (Sn), *S. officinalis* (So), *S. pratensis* (Sp), *S. sclarea* (Ss), and *S. verticillata* (Sv), as well as rosmarinic acid (R), trolox (T), and EDTA obtained for (**a**) DPPH scavenging activity, (**b**) NO scavenging activity, (**c**) reducing power, (**d**) iron chelating activity, (**e**) lipid peroxidation inhibition. The data are expressed as mean values of three independent experiments ± standard deviation and different small letters indicate a statistically significant difference (Tukey’s multiple comparisons test at 95% confidence level).

Various mechanisms of action can be simultaneously involved in the antioxidant effects of complex plant extracts, such as free radical scavenging, termination of oxidative chain reactions, reducing capacity, and binding of prooxidant metal ions [55,56]. Therefore, in our study, five different assays were employed to evaluate the antioxidant properties of the ethanolic extracts of selected *Salvia* species as well as to elucidate their mode of action. The most used antioxidant method, the DPPH assay, allowed us to determine the ability of plant extracts to act as free radical scavengers. DPPH is a stable free organic radical which can be neutralized in the presence of an antioxidant, via electron or hydrogen atom transfer, resulting in a colour change that can be easily monitored spectrophotometrically. All tested *Salvia* extracts demonstrated a concentration-dependent free radical scavenging ability (Supplementary Materials Table S1). To compare the antioxidant properties of these sage plants, the concentrations of extracts required to reduce the initial radical concentration by 50% (IC_50_) were determined, and the results are given in Figure 1. A lower IC_50_ value indicates a higher ability of the sample to act as an antioxidant. All tested *Salvia* species showed a high potency of free radical scavenging. According to the determined IC_50_ values (2.49–7.71 µg/mL), the antioxidant efficiency order was *S. verticillata* > *S. fruticosa* > *S. officinalis* > *S. nemorosa* > *S. glutinosa* > *S. pratensis* > *S. sclarea*. DPPH scavenging activity was the focus of previous antioxidant studies of sage plants [35,39,40,44,47,49,57,58,59]. Although all studies revealed the antiradical potential of *Salvia* species, large variations in testing conditions, type of extracts, and reported results were found. Our study pointed out that *S. verticillata* ethanolic extract demonstrated the strongest antioxidative properties equal to Trolox (IC_50_ = 2.50 µg/mL). However, Hanganu et al. [47] reported significantly higher concentrations of the *S. verticillata* ethanolic extract required for a 50% inhibition (42.92 µg/mL). *S. nemorosa, S. pratensis,* and *S. sclarea* also demonstrated much stronger scavenging activity than that reported in the above-mentioned study. In addition, our results on the DPPH scavenging activity of *S. glutinosa* and *S. officinalis* ethanolic extracts were comparable to earlier reports on IC_50_ values of methanolic extracts (3.2 µg/mL and 3.37 µg/mL, respectively) [35,44]. On the contrary, Brindisi et al. [60] reported much higher concentrations of *S. officinalis* methanolic extract required to scavenge 50% of the initial DPPH radicals (10.3–12.4 µg/mL). Tzanova et al. [61] indicated significantly lower antiradical activity of *S. sclarea* in comparison to *S. verticillata,* which is in accordance with our findings. Katanić Stanković et al. [49] reported that *S. verticillata* methanolic extract achieved an IC_50_ value of 33.04 µg/mL, while our study demonstrated tenfold stronger antioxidant activity of the Croatian specimen. Overall, our results reveal that the *Salvia* extracts can scavenge free radicals due to their hydrogen atom- and/or the electron-donating ability and, therefore, may be able to suppress initiation and/or propagation of free radical-mediated chain reactions. Considering that polyphenols are the main compounds with antioxidant properties in plant extracts, the Pearson correlation coefficient (*r*) was calculated to assess the relationship between the content of polyphenols and the antioxidant effect of the studied sage plants (Supplementary Materials Table S8). The correlation analysis revealed a very strong correlation between free radical scavenging activity and total phenolic acid content in *Salvia* species (*r* = 0.9478). Our data also suggest that caffeic, ferulic, and rosmarinic acid also contribute significantly to the antiradical activity of the sage plants. These results agree with the previous report [47]. A strong positive correlation between the quercetin content and DPPH scavenging activity (*r* = 0.8958) was also observed.

Nitric oxide (NO) is a biologically relevant free radical which, when produced in excessive amounts, reacts with another free radical, the superoxide anion, and forms highly reactive nitrogen species. Therefore, testing the ability of natural compounds to directly scavenge NO is suggested as a promising pharmacological strategy for the prevention and treatment of pathological conditions associated with oxidative stress, such as inflammatory and neurodegenerative disorders [55]. All tested *Salvia* species demonstrated NO scavenging activity in a concentration-dependent manner (Supplementary Materials Table S2). At the concentration of 100 µg/mL, the extracts of all investigated *Salvia* species were capable of scavenging more than 55% of generated NO radicals. However, the selected *Salvia* species displayed a wide range of IC_50_ values, from 26.96 µg/mL to 101.73 µg/mL (Figure 1). The *S. officinalis* ethanolic extract exhibited the most effective NO radical scavenging ability, followed by the extracts of *S. sclarea*, *S. fruticosa,* and *S. pratensis* (29.10–45.49 µg/mL). Interestingly, the mentioned *Salvia* extracts demonstrated significantly stronger antioxidative properties than Trolox (IC_50_ = 66.69 µg/mL). In addition, equal effects to those of Trolox were observed for *S. verticillata* and *S. glutinosa*, which also indicated their exceptional antioxidant properties. Rosmarinic acid was found to be a very potent NO scavenger (13.44 µg/mL). According to our results (Supplementary Materials Table S8), there is a moderate correlation between the NO scavenging activity of tested extracts and rosmarinic acid content (*r* = 0.5789), as well as *p*-coumaric acid (*r* = 0.5127), apigenin (*r* = 0.6242) and luteolin (*r* = 0.5762) contents. As far as our literature survey could ascertain, the data presented here are the first record on the NO scavenging ability for most of the investigated *Salvia* species. Only one recent study on the antioxidant activity of *S. verticillata* reported that the methanolic extract of aerial parts possesses NO radical scavenging activity (IC_50_ = 73.12 μg/mL), corresponding to our results [49]. 

The antioxidant properties of *Salvia* species in comparison with rosmarinic acid and Trolox were also evaluated by reducing the power assay. Antioxidants have an electron-donating ability and can cause the reduction of the Fe^3+^/ferricyanide complex to the ferrous form. The IC_50_ value provided absorbance of 0.5 and was calculated from the graph of absorbance against sample concentration [27]. All tested *Salvia* extracts were active in a concentration-dependent manner (Supplementary Materials Table S3). The absorbance values between 0.405 and 0.681 were determined for all samples at the concentration of 12.5 µg/mL. *S. officinalis*, S. *verticillata,* and *S. fruticosa,* containing the highest amounts of total phenolic acids, as well as rosmarinic acid (2.91–3.88%), were the most active sage plants. These findings can be correlated to the lowest IC_50_ values from 8.83 to 9.75 µg/mL. The rest of the tested *Salvia* species achieved IC_50_ values from 11.60 µg/mL to 17.51 µg/mL (Figure 1). Rosmarinic acid acted as a strong electron donor, demonstrating activity that was four times higher than the *Salvia* extracts with the best reducing power. The obtained results indicate a great contribution of rosmarinic and other phenolic acids to the reducing power capability of tested sage extracts. These observations were confirmed by Pearson’s coefficient (Supplementary Materials Table S8) which showed a strong correlation of reducing power and total phenolic acid content (*r* = 0.7784), as well as rosmarinic acid content (*r* = 0.7735). Our findings are in accordance with previous studies suggesting that the ability of different extracts of *S. fruticosa, S. officinalis*, *S. sclarea, and S. verticillata* to reduce Fe^3+^ are strongly correlated with caffeic acid derivatives contents [41,47,59]. On the other hand, the reported results for Libyan *S. fruticosa* indicate no correlation between caffeic acid content and reducing power [40]. Inconsistent findings could be attributed to the use of various plant samples, extraction solvents, and testing methods. 

The antioxidant properties of plant polyphenols are attributed to their ability to chelate transition metal ions which are known to catalyse an initial formation of reactive oxygen species [27]. In that context, the ability of selected *Salvia* species to chelate transition metal ions was tested by an iron-chelating activity assay with ferrozine as a competitive Fe^2+^ chelator. The ethanolic extracts of the *Salvia* species were tested in the concentration range from 100 to 1600 µg/mL, in comparison with EDTA as a reference chelating agent. All extracts achieved the efficiency of 52.57–83.99% at the highest tested concentration (Supplementary Materials Table S4). *S. sclarea* with an IC_50_ value of 163.02 µg/mL demonstrated the strongest chelating activity, while the highest IC_50_ values (1185.54–1582.53 µg/mL) were determined for *S. officinalis*, *S. verticillata*, and *S. fruticosa* (Figure 1). In contrast to *S. sclarea*, those three species, although rich in rosmarinic and total phenolic acids, showed weak chelating properties. A correlation between chelating ability and rosmarinic acid content as well as total phenolic acid content was not found (Supplementary Materials Table S8). Moreover, in this assay, rosmarinic acid demonstrated no activity at all, additionally indicating that the effectiveness of the tested *Salvia* extract could be attributed to flavonoids (*r* = 0.4752), individually apigenin (*r* = 0.8073), and luteolin (*r* = 0.8228). Our results confirm previous studies that showed weak chelating activities of *S. glutinosa*, *S*. *officinalis*, and *S. verticillata* [26,49,62]. On the contrary, one recent study demonstrated no chelating ability of *S. sclarea* even at the concentration of 2000 µg/mL [63]. 

Lipid peroxidation is a process in which oxidants attack lipids, especially polyunsaturated fatty acids. Under high lipid peroxidation rates, oxidative damage can overwhelm the cell repair capacity and cause various pathological conditions. One of the major toxic components generated by lipid peroxidation is malondialdehyde which can be spectrophotometrically detected by adding thiobarbituric acid [64]. The lipid peroxidation inhibition assay was conducted for selected *Salvia* species in comparison with rosmarinic acid. All tested ethanolic extracts inhibited lipid peroxidation in a concentration-dependent manner (Supplementary Materials Table S5). The ethanolic extract of *S. officinalis* was the most potent one (IC_50_ = 53.18 μg/mL) followed by *S. fruticosa*, *S. glutinosa*, *S. sclarea*, *S. verticillata*, *S. pratensis,* and *S. nemorosa*, with IC_50_ values from 116.83 μg/mL to 327.23 μg/mL (Figure 1). Obtained results accentuated rosmarinic acid as a potent inhibitor of lipid peroxidation and additionally confirmed its status as a significant antioxidant. A strong correlation (*r* = 0.7426) between rosmarinic acid content and inhibition of lipid peroxidation was confirmed. In addition, the content of rutin moderately correlated with the lipid peroxidation inhibitory activity (*r* = 0.5505) (Supplementary Materials Table S8). Our findings are consistent with previous investigations of *S. officinalis* from Algeria, whose ethanolic extract showed similar inhibition of lipid peroxidation [36]. In addition, the aqueous extract of *S. officinalis* ‘Icterina’ also demonstrated a strong inhibitory property [65]. The methanolic extract of *S. verticillata* from Serbia was found to be more potent than the ethanolic extract of *S. verticillata* tested in our study [49]. Moreover, in contrast to our results, the study performed by Zupkó et al. [34] reported much lower IC_50_ values for methanolic leaf extracts of *S. glutinosa*, *S. nemorosa*, *S. officinalis,* and *S. pratensis*, probably due to the differences in experimental procedures.

### 3.3. Acetylcholinesterase and α-Glucosidase Inhibitory Activities of Selected Salvia Species

Alzheimer’s disease (AD) is an irreversible neurodegenerative disorder characterised by a lack of neurotransmitter acetylcholine which has a key role in cognitive functions. Currently, the most prescribed drugs for AD treatment are cholinesterase inhibitors. In traditional European medicine, *Salvia* species are recognised for their memory-enhancing abilities [45,62,66]; so, within our study, we evaluated the acetylcholinesterase (AChE) inhibitory activity of selected *Salvia* species. Among tested *Salvia* species, only three of them were found to possess a certain capability to inhibit AChE (Table 3, Supplementary Materials Table S6). At the concentration of 400 µg/mL, *S. fruticosa* and *S. officinalis* inhibited 80% of the AChE enzyme activity. There was no statistical difference between their IC_50_ values (287.02 and 268.45 µg/mL, respectively) which were slightly higher than that of rosmarinic acid. *S. verticillata* achieved its IC_50_ value almost at the highest tested concentration of 1600 µg/mL. *S. glutinosa*, *S. nemorosa*, *S. sclarea,* and *S. pratensis* did not reach a 50% efficacy in the tested concentration range. Observed enzyme inhibitory activities strongly correlated with the presence of rosmarinic acid (*r* = 0.8168), *p*-coumaric acid (*r* = 0.8727), and luteolin-7-O-glucoside (*r* = 0.7050) in *Salvia* ethanolic extracts (Supplementary Materials Table S8). Our findings are consistent with other reports that also highlighted a significant contribution of rosmarinic acid to the neuroprotective properties of *S. officinalis* [50]. Bahadori et al. [45] reported that *S. nemorosa* methanolic, dichloromethane and hexane extracts showed 50% inhibitory activities in the range of 223.4–469.9 µg/mL which was not confirmed by our research. A previous report by Demirezer et al. [63,66] indicated significantly better AChE inhibitory activity of *S. fruticosa* in comparison to *S. verticillata* that we demonstrated in our study. Furthermore, literature data also reveal a low or non-existent AChE inhibitory potential of *S. sclarea, S. glutinosa,* and *S. verticillata* [63,67]. 

Diabetes is a complex progressive metabolic disorder that occurs due to insulin secretion deficiencies associated with high blood glucose levels. Chronic hyperglycemia is associated with long-term damage and dysfunction of many organs. Previous studies suggested that certain plant extracts may have beneficial effects on diabetes. Moreover, the history of some antidiabetic drugs such as metformin is linked to the traditional use of medicinal plants [2,67]. The strategy of reducing carbohydrate digestibility by controlling the activity of hydrolyzing enzymes of the small intestine is considered a viable prophylactic treatment of hyperglycemia. Acarbose is classified as an α-glucosidase inhibitor and is often used as a positive control in antidiabetic research. Synthetic antidiabetic drugs are usually associated with gastrointestinal side effects. Therefore, numerous studies have been conducted to identify enzyme inhibitors from natural sources. Various *Salvia* species have shown the great potential to be explored as an alternative strategy in diabetic therapies [67,68]. In view of all of the above, we evaluated the α-glucosidase inhibitory activity of selected *Salvia* species. Although all sage extracts in the tested concentrations (400–6400 µg/mL) showed the ability to inhibit the enzyme (Supplementary Materials Table S7), IC_50_ values were detected only for *S. fruticosa*, *S. officinalis,* and *S. glutinosa*. ranging from 4451 µg/mL to 5291 µg/mL. As it can be seen from Table 3, these values were four to five times higher than the IC_50_ value determined for acarbose. *S. officinalis* and *S. fruticosa* were the most potent enzyme inhibitors and their effects did not differ statistically from that of rosmarinic acid. Mocan et al. [50] also found that these two species equally inhibited α-glucosidase. Mahdi et al. [37] revealed that the ethyl acetate fraction of *S. officinalis* hydro-methanol decoction extract had the best antidiabetic activity tested both by α-amylase and α-glucosidase assays, which corresponded to the highest content of phenolic compounds. In this study, we show that tannins contributed the most to the observed enzyme inhibition activity of *Salvia* extracts which was indicated by the high correlation coefficient (*r* = 0.7934). A moderate correlation was also observed between the content of luteolin-7-O-glucoside and the α-glucosidase inhibitory activity in tested sage plants (*r* = 0.5791) (Supplementary Materials Table S8). Contrary to our results, one study reported that *S. nemorosa* methanolic extract had an α-glucosidase inhibitory effect comparable to acarbose [45]. Considering the above, our findings contributed to the elucidation of the antidiabetic potential of selected *Salvia* species by demonstrating their ability to inhibit α-glucosidase involved in carbohydrate digestion.

Recent evidence suggests that Alzheimer’s disease is closely related to impaired cerebral glucose metabolism and insulin resistance, as seen in type 2 diabetes. Insulin plays an important neuroprotective and trophic role in brain cells by preventing apoptosis, β-amyloid toxicity, and oxidative stress, as well as promoting neuronal survival and memory. Diabetes and neurodegenerative diseases have been found to share some insulin resistance-related mechanisms with oxidative stress as a pathogenic background [69,70]. In this context, our findings reveal that polyphenol-rich *Salvia* ethanolic extracts have the potential to prevent and mitigate neurodegenerative changes in diabetes by reducing oxidative stress and inhibiting enzymes involved in glucose metabolism and acetylcholine loss.

## 4. Conclusions

This study provides evidence of the antioxidant ability, AChE, and α-glucosidase inhibitory activities of wild-growing *Salvia* species from the Mediterranean region. To our knowledge, this is the first comparative study of selected *Salvia* species in terms of the biological activities tested. Moreover, some data on certain species were reported for the first time. All tested plants were able to scavenge free radicals, act as reducing agents, chelate transition metals, and inhibit lipid peroxidation as well as AChE and α-glucosidase. Together with *S. fruticosa* and *S. officinalis*, *S. verticillata,* and *S. glutinosa*, respectively, were the most effective antioxidants as well as AChE and α-glucosidase inhibitors. The observed biological activities of *Salvia* species were positively influenced mainly by total phenolic acids, total tannins, and rosmarinic acid, which was identified as the most abundant one (9400–38,800 μg/g). Other phenolic acids, such as caffeic, chlorogenic, *p*-coumaric, and ferulic acid, were also predominant in sage leaves (300–11,600 μg/g), in contrast to flavonoids, which were significantly less represented (10.82–306.81 μg/g). Luteolin, apigenin, quercetin, and their glycosidic derivatives were identified in the sage extracts; however, their content and composition varied significantly between tested specimens. Overall, our results reveal *Salvia* ethanolic extracts as a valuable source of polyphenols which are potent antioxidants with a multitarget mechanism of action, as well as moderate AChE and α-glucosidase inhibitors. Therefore, they can be considered as promising therapeutic agents for oxidative stress-related chronic diseases, such as diabetes, cardiovascular and neurodegenerative diseases, and serve as nutraceuticals and food preservatives.

## Figures and Tables

**Table 1:** Content of total flavonoids, phenolic acids, tannins, and anthocyanins (%) in the leaves of selected *Salvia* species.

Sample	Flavonoids	Phenolic Acids	Tannins	Anthocyanins
*S. fruticosa*	0.31 ± 0.009 ^g^	7.05 ± 0.13 ^c^	1.80 ± 0.08 ^b^	0.02 ± 0.001 ^f^
*S. glutinosa*	1.07 ± 0.002 ^a^	6.95 ± 0.22 ^c^	2.60 ± 0.08 ^a^	0.08 ± 0 ^a^
*S. nemorosa*	0.88 ± 0.004 ^c^	6.48 ± 0.01 ^d^	1.40 ± 0.12 ^c^	0.06 ± 0.002 ^b^
*S. officinalis*	0.37 ± 0.002 ^f^	8.04 ± 0.10 ^b^	1.83 ± 0.04 ^b^	0.03 ± 0.002 ^e^
*S. pratensis*	0.90 ± 0.007 ^b^	6.49 ± 0.01 ^d^	1.37 ± 0.13 ^c^	0.05 ± 0.002 ^c^
*S. sclarea*	0.75 ± 0.002 ^d^	3.55 ± 0.04 ^e^	1.22 ± 0.04 ^c^	0.05 ± 0 ^c^
*S. verticillata*	0.39 ± 0.003 ^e^	12.44 ± 0.01 ^a^	1.67 ± 0.09 ^b^	0.04 ± 0.002 ^d^

The data are expressed as mean values of three independent experiments ± standard deviation. Mean values displaying different letters within each column are significantly different according to Tukey’s multiple comparisons test at a 95% confidence level.

**Table 2:** Content of phenolic acids and flavonoids (μg/g of herbal material) of selected *Salvia* species determined by the HPLC-DAD method.

Compound	*S. fruticosa*	*S. glutinosa*	*S. nemorosa*	*S. officinalis*	*S. pratensis*	*S. sclarea*	*S. verticillata*
Phenolic acids							
Caffeic acid	1300 ± 10 ^b^	300 ± 0 ^f^	300 ± 10 ^f^	400 ± 10 ^e^	600 ± 10 ^c^	500 ± 20 ^d^	4100 ± 40 ^a^
Chlorogenic acid	300 ± 10 ^f^	300 ± 10 ^f^	1900 ± 10 ^b^	2100 ± 30 ^a^	1600 ± 10 ^c^	1200 ± 40 ^e^	1500 ± 30 ^d^
*p*-Coumaric acid	9000 ± 130 ^b^	1200 ± 10 ^e^	1600 ± 20 ^e^	11,600 ± 500 ^a^	600 ± 10 ^f^	2900 ± 20 ^d^	8400 ± 120 ^c^
Ferulic acid	1400 ± 20 ^b^	n.d.	n.d.	n.d.	n.d.	n.d.	2200 ± 30 ^a^
Rosmarinic acid	29,100 ± 210 ^c^	9400 ± 70 ^g^	14,200 ± 110 ^f^	38,800 ± 270 ^a^	19,500 ± 120 ^d^	17,900 ± 130 ^e^	30,200 ± 210 ^b^
Flavonoids							
Apigenin	10.82 ± 0.05 ^c^	n.d.	n.d.	13.48 ± 0.11 ^b^	n.d.	83.68 ± 0.25 ^a^	n.d.
Apigenin-7-O-glc	n.d.	156.83 ± 0.21	n.d.	n.d.	n.d.	n.d.	n.d.
Luteolin	11.86 ± 0.03 ^e^	n.d.	n.d.	27.51 ± 0.14 ^c^	25.62 ± 0.09 ^d^	163.54 ± 0.19 ^a^	31.48 ± 0.14 ^b^
Luteolin-7-O-glc	306.81 ± 0.14 ^a^	51.73 ± 0.12 ^c^	n.d.	55.74 ± 0.21 ^b^	n.d.	n.d.	n.d.
Quercetin	21.77 ± 0.10 ^b^	n.d.	n.d.	n.d.	n.d.	n.d.	59.02 ± 0.18 ^a^
Rutin	n.d.	n.d.	n.d.	75.28 ± 0.10 ^b^	76.24 ± 0.11 ^a^	n.d.	n.d.

The data are expressed as mean values of three independent experiments ± standard deviation. Mean values displaying different letters within each row are significantly different according to Tukey’s multiple comparisons test at a 95% confidence level. n.d.: not detected.

**Table 3:** Antiacetylcholinesterase and anti-α-glucosidase activities (IC_50_ values, µg/mL) of the ethanolic extracts of selected *Salvia* species, rosmarinic acid, and reference compounds.

Sample	Acetylcholinesterase Inhibition	α-Glucosidase Inhibition
*S. fruticosa*	287.02 ± 6.94 ^b^	5291.51 ± 335.08 ^a^
*S. glutinosa*	n.d.	4496.06 ± 66.36 ^b^
*S. nemorosa*	n.d.	n.d.
*S. officinalis*	268.45 ± 14.14 ^b^	4451.85 ± 142.22 ^b^
*S. pratensis*	n.d.	n.d.
*S. sclarea*	n.d	n.d.
*S. verticillata*	1607.87 ± 15.05 ^a^	n.d.
rosmarinic acid	234.77 ± 14.77 ^c^	4927.45 ± 324.81 ^b^
galantamine	0.12 ± 0.01 ^d^	-
acarbose	-	1104.76 ± 34.80 ^c^

The data are expressed as mean values of three independent experiments ± standard deviation. Mean values displaying different letters within each column are significantly different according to Tukey’s multiple comparisons test at a 95% confidence level. n.d.: not determined, -: not tested.

## Data Availability

All data that support the findings of this study are available in the Supplementary Materials of this article.

## Supplementary Materials

The following supporting information can be downloaded at: https://www.mdpi.com/article/10.3390/plants11050625/s1, Table S1: DPPH radical scavenging activity (%) of selected *Salvia* species in comparison with rosmarinic acid and a reference antioxidant; Table S2: NO free radical scavenging activity (%) of selected *Salvia* species in comparison with rosmarinic acid and a reference antioxidant; Table S3: Reducing power of selected *Salvia* species in comparison with rosmarinic acid and a reference antioxidant; Table S4: Iron(II) ions chelating activity (%) of selected *Salvia* species in comparison with rosmarinic acid and a reference chelator; Table S5: Inhibition of lipid peroxidation (%) of selected *Salvia* species in comparison with rosmarinic acid; Table S6: Inhibition of acetylcholinesterase (%) of selected *Salvia* species in comparison with rosmarinic acid and galantamine; Table S7: Inhibition of α-glucosidase (%) determined in the ethanolic extracts of selected *Salvia* species in comparison with acarbose, Table S8: The Pearson’s correlation coefficients between biological activities and polyphenolic contents of selected *Salvia* species.

## References

[1] Walker J.B., Sytsma K.J. (2007). Staminal evolution in the genus *Salvia* (Lamiaceae): Molecular phylogenetic evidence for multiple origins of the staminal lever. Ann. Bot..

[2] Rashed A.A., Gunasegavan Rathi D.N. (2021). Bioactive components of *Salvia* and their potential antidiabetic properties: A review. Molecules.

[3] Askari S.F., Avan R., Tayarani-Najaran Z., Sahebkar A., Eghbali S. (2021). Iranian *Salvia* species: A phytochemical and pharmacological update. Phytochemistry.

[4] Tulukcu E., Cebi N., Sagdic O. (2019). Chemical fingerprinting of seeds of seeds of some *Salvia* species in Turkey by using GC-MS and FTIR. Foods.

[5] Lopresti A.L. (2017). *Salvia* (Sage): A review of its potential cognitive-enhancing and protective effects. Drugs R D.

[6] Lu Y., Foo L.Y. (2002). Polyphenolics of *Salvia*—A review. Phytochemistry.

[7] Bonesi M., Loizzo M.R., Acquaviva R., Malfa G.A., Aiello F., Tundis R. (2017). Anti-inflammatory and antioxidant agents from *Salvia* genus (Lamiaceae): An assessment of the current state of knowledge. Antiinflamm. Antiallergy Agents Med. Chem..

[8] Afonso A.F., Pereira O.R., Cardoso S.M. (2021). *Salvia* species as nutraceuticals: Focus on antioxidant, antidiabetic and anti-obesity properties. Appl. Sci..

[9] Ververis A., Savvidou G., Ioannou K., Nicolaou P., Christodoulou K., Plioukas M. (2020). Greek sage exhibits neuroprotective activity against amyloid beta-induced toxicity. Evid. Based Complement. Altern. Med..

[10] Hao D.C., Ge G.B., Xiao P.G. (2018). Anticancer drug targets of *Salvia* phytometabolites: Chemistry, biology and omics. Curr. Drug Targets.

[11] Flora Croatica Database. https://hirc.botanic.hr/fcd/.

[12] Craft J.D., Satyal P., Setzer W.N. (2017). The chemotaxonomy of common sage (*Salvia officinalis*) based on the volatile constituents. Medicines.

[13] Tursun A.O., Sipahioglu H.M., Telci I. (2021). Genetic relationships and diversity within cultivated accessions of *Salvia officinalis* L. in Turkey. Plant Biotechnol. Rep..

[14] European Medicines Agency. https://www.ema.europa.eu.

[15] KammounEl Euch S., Hassine D.B., Cazaux S., Bouzouita N., Bouajila J. (2017). *Salvia officinalis* essential oil: Chemical analysis and evaluation of anti-enzymatic and antioxidant bioactivities. S. Afr. J. Bot..

[16] Ghorbani A., Esmaeilizadeh M. (2017). Pharmacological properties of *Salvia officinalis* and its components. J. Tradit. Complement. Med..

[17] Sabry M.M., Abdel-Rahman R., El-Shenawy S.M., Hassan A.M., El-Gayed S.H. (2022). Estrogenic activity of Sage (*Salvia officinalis* L.) aerial parts and its isolated ferulic acid in immature ovariectomized female rats. J. Ethnopharmacol..

[18] Tundis R., Leporini M., Bonesi M., Rovito S., Passalacqua N.G. (2020). *Salvia officinalis* L. from Italy: Comparative chemical and biological study of its essential oil in the Mediterranean context. Molecules.

[19] Montesino N.L., Kaiser M., Mäser P., Schmidt T.J. (2021). *Salvia officinalis* L.: Antitrypanosomal activity and active constituents against *Trypanosoma brucei rhodesiense*. Molecules.

[20] Sarikhan H., Tavan M., Rigano M.M., Azizi A. (2021). Triterpenic and phenolic acids production changed in *Salvia officinalis* via in vitro and in vivo polyploidization: A consequence of altered genes expression. Phytochemistry.

[21] Martins N., Barros L., Santos-Buelga C., Silva S., Ferreira I.C.F.R. (2015). Evaluation of bioactive properties and phenolic compounds in different extracts prepared from *Salvia officinalis* L.. Food Chem..

[22] Dent M., Bursač-Kovačević D., Bosiljkov T., Dragović-Uzelac V. (2017). Polyphenolic composition and antioxidant capacity of indigenous wild dalmatian sage (*Salvia officinalis* L). Croat. Chem. Acta.

[23] Velamuri R., Sharma Y., Fagan J., Schaefer J. (2020). Application of UHPLC-ESI-QTOF-MS in phytochemical profiling of sage (*Salvia officinalis*) and rosemary (*Rosmarinus officinalis*). Planta Med. Int. Open.

[24] European Pharmacopoeia Online. https://pheur.edqm.eu/home.

[25] Wagner H., Bladt S. (2009). Plant Drug Analysis.

[26] Bljajić K., Brajković A., Čačić A., Vujić L., Jablan J., de Carvalho I.S., Zovko Končić M. (2021). Chemical composition, antioxidant and α-glucosidase-inhibitory activity of aqueous and hydroethanolic extracts of traditional antidiabetics from Croatian ethnomedicine. Horticulturae.

[27] Vladimir-Knežević S., Blažeković B., Bival Štefan M., Alegro A., Köszegi T., Petrik J. (2011). Antioxidant activities and polyphenolic contents of three selected *Micromeria* species from Croatia. Molecules.

[28] Patel A., Patel A., Patel A., Patel N.M. (2010). Determination of polyphenols and free radical scavenging activity of *Tephrosia purpurea* Linn leaves (Leguminosae). Pharmacogn. Res..

[29] Benabdallah A., Rahmoune C., Boumendjel M., Aissi O., Messaoud C. (2016). Total phenolic content and antioxidant activity of six wild *Mentha* species (Lamiaceae) from northeast of Algeria. Asian Pac. J. Trop. Biomed..

[30] Houghton P.J., Zarka R., de las Heras B., Hoult J.R. (1995). Fixed oil of *Nigella sativa* and derived thymoquinone inhibit eicosanoid generation in leukocytes and membrane lipid peroxidation. Planta Med..

[31] Conforti F., Statti G.A., Tundis R., Loizzo M.R., Menichini F. (2007). In vitro Activities of *Citrus medica* L. cv. Diamante (Diamante citron) relevant to treatment of diabetes and Alzheimer’s disease. Phytother. Res..

[32] Bljajić K., Petlevski R., Vujić L., Čačić A., Šoštarić N., Jablan J., Saraiva de Carvalho I., Zovko Končić M. (2017). Chemical composition, antioxidant and α-glucosidase inhibiting activities of the aqueous and hydroethanolic extracts of *Vaccinium myrtillus* leaves. Molecules.

[33] Vladimir-Knežević S., Blažeković B., Kindl M., Vladić J., Lower-Nedza A.D., Brantner A.H. (2014). Acetylcholinesterase inhibitory, antioxidant and phytochemical properties of selected medicinal plants of the Lamiaceae family. Molecules.

[34] Zupkó I., Hohmann J., Rédei D., Falkay G., Janicsák G., Máthé I. (2001). Antioxidant activity of leaves of *Salvia* species in enzyme-dependent and enzyme-independent systems of lipid peroxidation and their phenolic constituents. Planta Med..

[35] Farhat M.B., Landoulsi A., Chaouch-Hamada R., Sotomayor J.A., Jordán M.J. (2013). Characterization and quantification of phenolic compounds and antioxidant properties of *Salvia* species growing in different habitats. Ind. Crops Prod..

[36] Boufadi A.M.Y., Keddari S., Moulai-Hacene F., Chaa S. (2021). Chemical composition, antioxidant and anti-inflammatory properties of *Salvia officinalis* extract from Algeria. Pharmacogn. J..

[37] Mahdi S., Rachid A., Lahfa B.F. (2020). Evaluation of in vitro α-amylase and α-glucosidase inhibitory potential and hemolytic effect of phenolic enriched fractions of the aerial part of *Salvia officinalis* L.. Diabetes Metab. Syndr. Clin. Res. Rev..

[38] Jeshvaghani Z.A., Rahimmalek M., Talebi M., Goli S.A.H. (2015). Comparison of total phenolic content and antioxidant activity in different *Salvia* species using three model systems. Ind. Crops Prod..

[39] Dincer C., Topuz A., Sahin-Nadeem H., Ozdemir K.S., Cam I.B., Tontul I., Gokturk R.S., Ay S.T. (2012). A comparative study on phenolic composition, antioxidant activity and essential oil content of wild and cultivated sage (*Salvia fruticosa* Miller) as influenced by storage. Ind. Crops Prod..

[40] Duletić-Laušević S., Alimpić Aradski A., Šavikin K., Knežević A., Milutinović M., Stević T., Vukojević J., Marković S., Marin P.D. (2018). Composition and biological activities of Libyan *Salvia fruticosa* Mill. and *S. lanigera* Poir. extracts. S. Afr. J. Bot..

[41] Vergine M., Nicolì F., Negro C., Luvisi A., Nutricati E., Annunziata Accogli R., Sabella E., Miceli A. (2019). Phytochemical profiles and antioxidant activity of *Salvia* species from southern Italy. Rec. Nat. Prod..

[42] Stagos D., Portesis N., Spanou C., Mossialos D., Aligiannis N., Chaita E., Panagoulis C., Reri E., Skaltsounis L.A., Tsatsakis A.M. (2012). Correlation of total polyphenolic content with antioxidant and antibacterial activity of 24 extracts from Greek domestic Lamiaceae species. Food Chem. Toxicol..

[43] Şenol F.S., Orhan I.E., Celep F., Kahraman A., Doǧan M., Yılmaz G., Şener B. (2010). Survey of 55 Turkish *Salvia* taxa for their acetylcholinesterase inhibitory and antioxidant activities. Food Chem..

[44] Loizzo M.R., Abouali M., Salehi P., Sonboli A., Kanani M., Menichini F., Tundis R. (2014). In vitro antioxidant and antiproliferative activities of nine *Salvia* species. Nat. Prod. Res..

[45] Bahadori M.B., Asghari B., Dinparast L., Zengin G., Sarikurkcu C., Abbas-Mohammadi M., Bahadori S. (2017). *Salvia nemorosa* L.: A novel source of bioactive agents with functional connections. LWT-Food Sci. Technol..

[46] Tosun M., Ercisli S., Sengul M., Ozer H., Polat T., Ozturk E. (2009). Antioxidant properties and total phenolic content of eight *Salvia* species from Turkey. Biol. Res..

[47] Hanganu D., Olah N.K., Pop C.E., Vlase L., Oniga I., Ciocarlan N., Matei A., Puşcaş C., Silaghi-Dumitrescu R., Benedec D. (2019). Evaluation of the polyphenolic profile and antioxidant activity for some *Salvia* species. Farmacia.

[48] Asadi S., Ahmadiani A., Esmaeili M.A., Sonboli A., Ansari N., Khodagholi F. (2010). In vitro antioxidant activities and an investigation of neuroprotection by six *Salvia* species from Iran: A comparative study. Food Chem. Toxicol..

[49] Katanić Stanković J.S., Srećković N., Mišić D., Gašić U., Imbimbo P., Monti D.M., Mihailović V. (2020). Bioactivity, biocompatibility and phytochemical assessment of lilac sage, *Salvia verticillata* L. (Lamiaceae)—A plant rich in rosmarinic acid. Ind. Crops Prod..

[50] Mocan A., Babotă M., Pop A., Fizes I., Diuzheva A., Locatelli M., Carradori S., Campestre C., Menghini L., Sisea C.R. (2020). Chemical constituents and biologic activities of sage species: A comparison between *Salvia officinalis* L., *S. glutinosa* L. and *S. transsylvanica* (Schur ex Griseb.& Schenk) Schur. Antioxidants.

[51] Sarrou E., Martens S., Chatzopoulou P. (2016). Metabolite profiling and antioxidative activity of Sage (*Salvia fruticosa* Mill.) under the influence of genotype and harvesting period. Ind. Crops Prod..

[52] Zengin G., Senkardes I., Mollica A., Picot-Allain C.M.N., Bulut G., Dogan A., Mahomoodally M.F. (2018). New insights into the in vitro biological effects, in silico docking and chemical profile of clary sage—*Salvia sclarea* L.. Comput. Biol. Chem..

[53] Cvetkovikj I., Stefkov G., Acevska J., Stanoeva J.P., Karapandzova M., Stefova M., Dimitrovska A., Kulevanova S. (2013). Polyphenolic characterization and chromatographic methods for fast assessment of culinary *Salvia* species from South East Europe. J. Chromatogr. A.

[54] Šulniūte V., Pukalskas A., Venskutonis P.R. (2017). Phytochemical composition of fractions isolated from ten *Salvia* species by supercritical carbon dioxide and pressurized liquid extraction methods. Food Chem..

[55] Kindl M., Blažeković B., Bucar F., Vladimir-Knežević S. (2015). Antioxidant and anticholinesterase potential of six *Thymus* species. Evid. Based Complement. Altern. Med..

[56] Schlesier K., Harwat M., Böhm V., Bitsch R. (2002). Assessment of antioxidant activity by using different in vitro methods. Free Radic. Res..

[57] Orhan I., Kartal M., Naz Q., Ejaz A., Yilmaz G., Kan Y., Konuklugil B., Şener B., Iqbal Choudhary M. (2007). Antioxidant and anticholinesterase evaluation of selected Turkish *Salvia* species. Food Chem..

[58] Boukhary R., Raafat K., Ghoneim A.I., Aboul-Ela M., El-Lakany A. (2016). Anti-inflammatory and antioxidant activities of *Salvia fruticosa*: An HPLC determination of phenolic contents. Evid. Based Complement. Altern. Med..

[59] Pereira O.R., Catarino M.D., Afonso A.F., Silva A.M.S., Cardoso S.M. (2018). *Salvia elegans*, *Salvia greggii* and *Salvia officinalis* decoctions: Antioxidant activities and inhibition of carbohydrate and lipid metabolic enzymes. Molecules.

[60] Brindisi M., Bouzidi C., Frattaruolo L., Loizzo M.R., Cappello M.S., Dugay A., Deguin B., Lauria G., Cappello A.R., Tundis R. (2021). New insights into the antioxidant and anti-inflammatory effects of Italian *Salvia officinalis* leaf and flower extracts in lipopolysaccharide and tumor-mediated inflammation models. Antioxidants.

[61] Tzanova M.T., Gerdzhikova M.A., Grozeva N.H., Terzieva S.R. (2019). Antioxidant activity and total phenolic content of five *Salvia* species from Bulgaria. Bulg. Chem. Commun..

[62] Orhan I.E., Sezer Şenol F., Ercetin T., Kahraman A., Celep F., Akaydin G., Şener B., Doğan M. (2013). Assessment of anticholinesterase and antioxidant properties of selected sage (*Salvia*) species with their total phenol and flavonoid contents. Ind. Crops Prod..

[63] Ekin H.N., Deliorman Orhan D., Erdoğan Orhan I., Orhan N., Aslan M. (2019). Evaluation of enzyme inhibitory and antioxidant activity of some Lamiaceae plants. J. Res. Pharm..

[64] Ayala A., Muñoz M.F., Argüelles S. (2014). Lipid peroxidation: Production, metabolism, and signalling mechanisms of malondialdehyde and 4-hydroxy-2-nonenal. Oxid. Med. Cell. Longev..

[65] Afonso A.F., Pereira O.R., Fernandes Â., Calhelha R.C., Silva A.M.S., Ferreira I.C.F.R., Cardoso S.M. (2019). Phytochemical composition and bioactive effects of *Salvia africana*, *Salvia officinalis* ‘Icterina’ and *Salvia mexicana* aqueous extracts. Molecules.

[66] Demirezer L.Ö., Gürbüz P., Kelicen Uğur E.P., Bodur M., Özenver N., Uz A., Güvenalp Z. (2015). Molecular docking and ex vivo and in vitro anticholinesterase activity studies of *Salvia* sp. and highlighted rosmarinic acid. Turk. J. Med. Sci..

[67] El-Tantawy W.H., Temraz A. (2018). Management of diabetes using herbal extracts: Review. Arch. Physiol. Biochem..

[68] Assefa A.T., Yang E.-Y., Chae S.-O., Song M., Lee M., Cho M.-C., Jang S. (2020). Alpha glucosidase inhibitory activities of plants with focus on common vegetables. Plants.

[69] Berlanga-Acosta J., Guillén-Nieto G., Rodríguez-Rodríguez N., Bringas-Vega M.L., García-Del-Barco-Herrera D., Berlanga-Saez J.O., García-Ojalvo A., Valdés-Sosa M.J., Valdés-Sosa P.A. (2020). Insulin resistance at the crossroad of Alzheimer disease pathology: A review. Front. Endocrinol..

[70] Butterfield D.A., Halliwell B. (2019). Oxidative stress, dysfunctional glucose metabolism and Alzheimer disease. Nat. Rev. Neurosci..

