# Supplementary material for: Comparative Antioxidant, Anti-Acetylcholinesterase and Anti-α-Glucosidase Activities of Mediterranean Salvia Species
Source: Plants (Basel). 2022 Feb 25;11(5):625. doi: 10.3390/plants11050625 (PMC8912324; doi:10.3390/plants11050625)
Supplement: Supplementary file 1 [file plants-11-00625-s001.zip › Supplement_Table S1_Mervic et al. Salvia species.pdf]

Table S1. DPPH radical scavenging activity (%) of selected *Salvia* species in comparison with rosmarinic acid and a reference antioxidant.

Sample	0.78 μg/mL	1.56 μg/mL	3.13 μg/mL	6.25 μg/mL	12.5 μg/mL	25 μg/mL	50 μg/mL
S. fruticosa	10.51 ± 5.13^{c}	26.81 ± 2.56^{c,d}	51.09 ± 0.52^{b}	79.17 ± 0.77^{a}	79.53 ± 1.28^{c}	82.07 ± 0.26^{a}	82.05 ± 1.77^{b}
S. glutinosa	6.20 ± 0.64^{c}	16.77 ± 2.35^{e}	33.24 ± 0.86^{d}	64.20 ± 2.35^{d}	76.89 ± 0.21^{d}	78.10 ± 0.64^{b}	75.08 ± 1.07^{d}
S. nemorosa	10.07 ± 0.98^{c}	20.49 ± 1.97^{e}	39.03 ± 3.63^{c,d}	69.78 ± 3.42^{c}	80.70 ± 0.28^{c}	83.15 ± 0.22^{a}	83.86 ± 0.25^{b}
S. officinalis	8.41 ± 1.04^{c}	21.68 ± 0.21^{d,e}	41.15 ± 2.29^{c}	74.78 ± 0.62^{a,b,c}	78.17 ± 0^{c,d}	79.36 ± 0.42^{b,c}	81.57 ± 0.21^{b}
S. pratensis	8.85 ± 1.67^{c}	16.96 ± 1.46^{e}	31.12 ± 1.88^{d}	64.16 ± 2.72^{d}	78.03 ± 1.04^{c,d}	77.29 ± 0^{b}	76.11 ± 0.42^{d}
S. sclarea	4.88 ± 0^{c}	8.89 ± 3.70^{f}	23.70 ± 3.94^{e}	43.21 ± 1.48^{e}	75.79 ± 0.25^{d}	80.31 ± 1.23^{c}	79.27 ± 0.25^{c}
S.verticillata	17.92 ± 1.24^{b}	30.37 ± 1.04^{b,c}	63.13 ± 1.80^{a}	77.60 ± 0.78^{a,b}	79.82 ± 0.26^{c}	79.82 ± 0.26^{c}	82.20 ± 0^{b}
Rosmarinic acid	37.75 ± 2.48^{a}	59.17 ± 0.38^{a}	63.31 ± 0.56^{a}	73.39 ± 1.12^{b,c}	92.09 ± 0.12^{a}	95.14 ± 0.33^{d}	95.32 ± 0.07^{a}
Trolox	15.32 ± 0.80^{b,c}	34.59 ± 1.44^{b}	61.44 ± 1.12^{a}	74.23 ± 0.19^{a,b,c}	89.55 ± 0.03^{b}	93.51 ± 0.02^{d}	94.59 ± 0.01^{a}

The data are expressed as mean values of three independent experiments ± standard deviation. Mean values displaying different letters within each column are significantly different according to the Tukey’s multiple comparisons test at 95% confidence level.
