# Supplementary material for: Comparative Antioxidant, Anti-Acetylcholinesterase and Anti-α-Glucosidase Activities of Mediterranean Salvia Species
Source: Plants (Basel). 2022 Feb 25;11(5):625. doi: 10.3390/plants11050625 (PMC8912324; doi:10.3390/plants11050625)
Supplement: Supplementary file 1 [file plants-11-00625-s001.zip › Supplement_Table S2_Mervic et al. Salvia species.pdf]

Table S2. NO free radical scavenging activity (%) of selected *Salvia* species in comparison with rosmarinic acid and a reference antioxidant.

Sample	6.25 μg/mL	12.5 μg/mL	25 μg/mL	50 μg/mL	100 μg/mL	200 μg/mL	400 μg/mL
S. fruticosa	5.16 ± 2.04[c]	22.97 ± 1.83[b,c,d]	48.16 ± 1.95[b]	51.81 ± 0.97[c]	66.48 ± 2.93[b]	80.21 ± 2.38[b]	82.30 ± 1.51[b]
S. glutinosa	16.73 ± 8.89[b,c]	28.01 ± 2.97[a,d]	31.58 ± 4.78[c,d]	47.51 ± 1.40[d,e]	61.60 ± 3.30[b,c]	68.67 ± 0.97[c]	67.22 ± 0.85[d,e]
S. nemorosa	11.45 ± 8.78[c]	20.72 ± 0.09[c,d]	28.23 ± 4.13[c]	44.07 ± 3.98[e]	56.08 ± 4.85[c,d,e]	62.19 ± 1.55[d]	63.62 ± 1.14[e]
S. officinalis	31.59 ± 1.95[a]	41.63 ± 3.44[a]	52.07 ± 2.16[b]	58.35 ± 0.36[b]	65.52 ± 1.10[b]	70.41 ± 0.38[c]	73.68 ± 0.34[c]
S. pratensis	20.81 ± 3.98[a]	37.30 ± 0.70[a,b]	48.35 ± 0.74[b]	50.03 ± 1.35[c,d]	55.71 ± 3.35[d,e]	58.77 ± 0.69[d]	64.28 ± 0.96[e]
S. sclarea	24.23 ± 2.33[a,b]	34.59 ± 10.19[a,c]	49.57 ± 0.03[b]	53.34 ± 1.06[c]	63.17 ± 0.13[b,e]	70.07 ± 1.80[c]	70.70 ± 1.72[c,d]
S. verticillata	5.94 ± 3.62[c]	28.55 ± 6.95[a]	41.67 ± 1.40[b,d]	42.83 ± 0.42[e]	65.04 ± 3.13[b]	80.17 ± 3.46[b]	81.55 ± 1.72[b]
Rosmarinic acid	30.66 ± 0.67[a]	55.30 ± 7.61[a]	71.11 ± 2.61[a]	76.80 ± 2.21[a]	81.63 ± 2.93[a]	84.07 ± 3.59[a,b]	85.13 ± 2.65[b]
Trolox	NA	25.90 ± 5.30[b]	36.35 ± 8.25[c]	42.64 ± 0.54[e]	63.37 ± 2.90[b,c]	88.79 ± 2.28[a]	91.82 ± 1.95[a]

The data are expressed as mean values of three independent experiments ± standard deviation. Mean values displaying different letters within each column are significantly different according to the Tukey's multiple comparisons test at 95% confidence level. NA: not active.
