# Supplementary material for: Comparative Antioxidant, Anti-Acetylcholinesterase and Anti-α-Glucosidase Activities of Mediterranean Salvia Species
Source: Plants (Basel). 2022 Feb 25;11(5):625. doi: 10.3390/plants11050625 (PMC8912324; doi:10.3390/plants11050625)
Supplement: Supplementary file 1 [file plants-11-00625-s001.zip › Supplement_Table S3_Mervic et al. Salvia species.pdf]

Table S3. Reducing power of selected *Salvia* species in comparison with rosmarinic acid and a reference antioxidant.

Sample	1.56 μg/mL	3.13 μg/mL	6.25 μg/mL	12.5 μg/mL	25 μg/mL	50 μg/mL	100 μg/mL
S. fruticosa	0.125 ± 0.001[d]	0.201 ± 0.011[d,e]	0,363 ± 0.038[c,d]	0.608 ± 0.017[d]	1.118 ± 0.091[c]	1.631 ± 0.086[d,e]	2.924 ± 0.066[b]
S. glutinosa	0.110 ± 0.001[e,f]	0.181 ± 0.001[e,f]	0.315 ± 0.004[e]	0.535 ± 0.004[e]	0.964 ± 0.080[d]	1.267 ± 0.100[f]	2.215 ± 0.021[c]
S. nemorosa	0.080 ± 0.001[g]	0.137 ± 0.004[g]	0.248 ± 0.010[f]	0.447 ± 0.005[f]	0.798 ± 0.031[e]	1.350 ± 0.028[f]	2.180 ± 0.180[c]
S. officinalis	0.139 ± 0.008[b,c]	0,232 ± 0.017[c]	0.375 ± 0.008[c]	0.681 ± 0.023[c]	1.156 ± 0.018[c]	1.839 ± 0.013[c]	3.099 ± 0.249[b]
S. pratensis	0.107 ± 0.008[f]	0.179 ± 0.002[f]	0.319 ± 0.013[d,e]	0.523 ± 0.022[e]	0.956 ± 0.008[d]	1.578 ± 0.025[e]	2.747 ± 0.040[b]
S. sclarea	0.087 ± 0.001[g]	0.143 ± 0.005[g]	0.243 ± 0.024[f]	0.405 ± 0.014[f]	0.659 ± 0.034[e]	1.115 ± 0.015[f]	1.657 ± 0.052[d]
S. verticillata	0.122 ± 0.003[d,e]	0.210 ± 0.001[d]	0.365 ± 0.010[c,d]	0.670 ± 0.003[c]	1.127 ± 0.062[c]	1.768 ± 0.096[c,d]	2.974 ± 0.065[b]
Rosmarinic acid	0.394 ± 0.004[a]	0.704 ± 0[a]	1.193 ± 0.004[a]	1.835 ± 0.021[a]	3.329 ± 0.030[a]	3.434 ± 0.002[a]	3.451 ± 0.007[a]
Trolox	0.151 ± 0.002[b]	0.271 ± 0.007[b]	0.488 ± 0.004[b]	0.897 ± 0.016[b]	1.466 ± 0.001[b]	2.952 ± 0.003[b]	3.424 ± 0.008[a]

The data are expressed as mean values of three independent experiments ± standard deviation. Mean values displaying different letters within each column are significantly different according to the Tukey’s multiple comparisons test at 95% confidence level.
