# Supplementary material for: Comparative Antioxidant, Anti-Acetylcholinesterase and Anti-α-Glucosidase Activities of Mediterranean Salvia Species
Source: Plants (Basel). 2022 Feb 25;11(5):625. doi: 10.3390/plants11050625 (PMC8912324; doi:10.3390/plants11050625)
Supplement: Supplementary file 1 [file plants-11-00625-s001.zip › Supplement_Table S4_Mervic et al. Salvia species.pdf]

Table S4. Iron(II) ions chelating activity (%) of selected *Salvia* species in comparison with rosmarinic acid and a reference chelator.

**Sample**	**100 μg/mL**	**200 μg/mL**	**400 μg/mL**	**800 μg/mL**	**1600 μg/mL**
S. fruticosa	0.38 ± 0.11^{b,c}	1.36 ± 1.06^{c}	1.66 ± 0.63^{g}	8.60 ± 1.95^{g}	52.57 ± 2.25^{d}
S. glutinosa	9.58 ± 0.13^{b}	22.63 ± 1.88^{b}	42.69 ± 1.86^{d}	56.10 ± 2.11^{d}	59.02 ± 1.39^{c}
S. nemorosa	3.81 ± 1.34^{b,c}	23.81 ± 0.21^{b}	55.61 ± 1.75^{c}	76.12 ± 1.15^{b}	83.99 ± 1.56^{a}
S. officinalis	NA	NA	6.31 ± 0.11^{f}	24.14 ± 0.44^{f}	75.23 ± 0^{b}
S. pratensis	5.86 ± 4.04^{b,c}	19.52 ± 6.80^{b,c}	61.71 ± 0.42^{b}	68.10 ± 1.80^{c}	74.70 ± 2.23^{b}
S. sclarea	30.70 ± 3.30^{a}	69.30 ± 4.55^{a}	83.40 ± 0.52^{a}	83.55 ± 0.94^{a}	83.71 ± 0.74^{a}
S. verticillata	2.90 ± 1.74^{c}	9.92 ± 3.50^{c}	26.83 ± 2.52^{e}	45.02 ± 1.21^{e}	54.36 ± 1.97^{d}
Rosmarinic acid	NA	NA	NA	NA	NA
**Sample**	**0.78 μg/mL**	**1.56 μg/mL**	**3.13 μg/mL**	**6.25 μg/mL**	**12.5 μg/mL**
EDTA	2.32 ± 1.24	12.78 ± 3.62	63.50 ± 1.02	97.28 ± 0.23	97.68 ± 0.34

The data are expressed as mean values of three independent experiments ± standard deviation. Mean values displaying different letters within each column are significantly different according to the Tukey's multiple comparisons test at 95% confidence level. NA: not active.
