# Supplementary material for: Comparative Antioxidant, Anti-Acetylcholinesterase and Anti-α-Glucosidase Activities of Mediterranean Salvia Species
Source: Plants (Basel). 2022 Feb 25;11(5):625. doi: 10.3390/plants11050625 (PMC8912324; doi:10.3390/plants11050625)
Supplement: Supplementary file 1 [file plants-11-00625-s001.zip › Supplement_Table S5_Mervic et al. Salvia species.pdf]

Table S5. Inhibition of lipid peroxidation (%) of selected *Salvia* species in comparison with rosmarinic acid.

Sample	12.5 μg/mL	25 μg/mL	50 μg/mL	100 μg/mL	200 μg/mL	400 μg/mL
S. fruticosa	-	-	35.72 ± 2.86[b]	52.01 ± 0.52[b]	62.49 ± 4.29[c,d]	77.94 ± 1.11[b]
S. glutinosa	-	-	6.07 ± 1.04[e]	46.20 ± 2.43[b,c]	70.49 ± 1.83[b]	73.69 ± 0.67[b,c]
S. nemorosa	-	-	19.23 ± 2.22[c]	24.63 ± 4.34[d]	39.60 ± 3.51[e]	56.82 ± 1.68[e]
S. officinalis	-	-	39.21 ± 0,91[b]	78.52 ± 2.82[a]	88.85 ± 1.94[a]	90.35 ± 1.38[a]
S. pratensis	-	-	3.22 ± 2.69[e]	20.77 ± 0.30[d]	57.88 ± 2.77[d]	74.17 ± 2.62[b,d]
S. sclarea	-	-	11.48 ± 0.35[d]	47.93 ± 1.93[b]	66.61 ± 1.05[b,c]	69.92 ± 1.41[c,d]
S. verticillata	-	-	13.22 ± 0.64[d]	37.53 ± 8.17[c]	62.58 ± 1.10[c]	74.16 ± 1.24[b,d]
Rosmarinic acid	27.63 ± 0.70	46.18 ± 3.23	67.05 ± 0.82[a]	74.15 ± 1.00[a]	-	-

The data are expressed as mean values of three independent experiments ± standard deviation. Mean values displaying different letters within each column are significantly different according to the Tukey's multiple comparisons test at 95% confidence level.
-: not tested
