# Supplementary material for: Comparative Antioxidant, Anti-Acetylcholinesterase and Anti-α-Glucosidase Activities of Mediterranean Salvia Species
Source: Plants (Basel). 2022 Feb 25;11(5):625. doi: 10.3390/plants11050625 (PMC8912324; doi:10.3390/plants11050625)
Supplement: Supplementary file 1 [file plants-11-00625-s001.zip › Supplement_Table S6_Mervic et al. Salvia species.pdf]

Table S6. Inhibition of acetylcholinesterase (%) of selected *Salvia* species in comparison with rosmarinic acid and galantamine.

Sample	100 μg/mL	200 μg/mL	400 μg/mL	800 μg/mL	1600 μg/mL
S. fruticosa	0.49 ± 0.35[c]	26.75 ± 2.35[c]	80.08 ± 2.09[b]	98.63 ± 1.17[a]	98.91 ± 1.06[a]
S. glutinosa	NA	NA	NA	NA	35.11 ± 0[d]
S. nemorosa	NA	NA	NA	NA	8.09 ± 1.04[f]
S. officinalis	NA	38.52 ± 11.84[b,c]	83.02 ± 0.33[b]	95.39 ± 2.91[a,b]	96,74 ± 1,90[a,b]
S. pratensis	NA	NA	NA	5.90 ± 1.85[c]	30.50 ± 5.10[d,e]
S. sclarea	NA	NA	NA	NA	27.45 ± 0.3[e]
S. verticillata	NA	NA	NA	9.18 ± 0.93[c]	51.30 ± 0.92[c]
Rosmarinic acid	8.67 ± 5.48[b]	47.22 ± 1.51[b]	92.95 ± 5.13[a]	92.96 ± 0.30[b]	93.25 ± 0.25[b]
Galantamine	100 ± 0[a]	100 ± 0[a]	100 ± 0[a]	100 ± 0[a]	100 ± 0[a]

The data are expressed as mean values of three independent experiments ± standard deviation. Mean values displaying different letters within each row are significantly different according to the Tukey's multiple comparisons test at 95% confidence level. NA: not active, -: not tested
