# Supplementary material for: Comparative Antioxidant, Anti-Acetylcholinesterase and Anti-α-Glucosidase Activities of Mediterranean Salvia Species
Source: Plants (Basel). 2022 Feb 25;11(5):625. doi: 10.3390/plants11050625 (PMC8912324; doi:10.3390/plants11050625)
Supplement: Supplementary file 1 [file plants-11-00625-s001.zip › Supplement_Table S7_Mervic et al. Salvia species.pdf]

Table S7. Inhibition of α-glucosidase (%) determined in the ethanolic extracts of selected *Salvia* species in comparison with acarbose.

Sample	400 μg/mL	800 μg/mL	1600 μg/mL	3200 μg/mL	6400 μg/mL
S. fruticosa	5.17 ± 3.86^{b}	9.14 ± 5.68^{b}	17.81 ± 4.15^{b}	32.54 ± 5.32^{c}	59.36 ± 1.86^{c}
S. glutinosa	4.60 ± 2.70^{b}	8.22 ± 2.20^{b}	$12.66 \pm 0.74^{b,d}$	30.63 ± 2.55^{c}	$75.95 \pm 2.89^{b,d}$
S. nemorosa	3.31 ± 2.49^{b}	6.15 ± 2.23^{b}	$6.43 \pm 1.40^{c,d}$	12.07 ± 1.59^{d}	39.32 ± 4.34^{e}
S. officinalis	3.56 ± 1.47^{b}	6.59 ± 0.89^{b}	$14.59 \pm 0.22^{b,e}$	44.03 ± 2.20^{b}	$68.57 \pm 1.03^{c,d}$
S. pratensis	3.21 ± 2.58^{b}	3.29 ± 2.58^{b}	$7.45 \pm 5.32^{c,e,f}$	15.70 ± 1.82^{d}	$32.37 \pm 6.86^{e,f}$
S. sclarea	3.20 ± 3.09^{b}	5.09 ± 1.82^{b}	$11.53 \pm 1.62^{b,f,g}$	$19.08 \pm 3.01^{d,e}$	43.03 ± 0.84^{e}
S. verticillata	4.91 ± 2.96^{b}	5.43 ± 3.81^{b}	$6.26 \pm 2.65^{c,g}$	8.12 ± 3.74^{d}	24.88 ± 4.09^{f}
rosmarinic acid	$1.89 + 1.53^{b}$	$6.82 + 0.81^{b}$	$13.51 + 4.67^{b}$	$27.94 + 5.75^{c,e}$	$67.48 + 2.11^{b,c}$
acarbose	26.75 ± 0.44^{a}	39.90 ± 3.73^{a}	66.31 ± 0.24^{a}	77.92 ± 0.12^{a}	89.13 ± 1.19^{a}

The data are expressed as mean values of three independent experiments ± standard deviation. Mean values displaying different letters within each row are significantly different according to the Tukey's multiple comparisons test at 95% confidence level.
