# Supplementary material for: Comparative Antioxidant, Anti-Acetylcholinesterase and Anti-α-Glucosidase Activities of Mediterranean Salvia Species
Source: Plants (Basel). 2022 Feb 25;11(5):625. doi: 10.3390/plants11050625 (PMC8912324; doi:10.3390/plants11050625)
Supplement: Supplementary file 1 [file plants-11-00625-s001.zip › Supplement_Table S8_Mervic et al. Salvia species.pdf]

Table S8. The Pearson's correlation coefficients between biological activities and polyphenolic contents of selected *Salvia* species.

	TF	TPA	TT	TA	RA	ChA	pCA	CA	FA	A	A-7-G	L	L-7-G	Q	R
**DPPH assay**	-0.6313^{a}	0.9478^{a}	0.1664^{a}	-0.4288^{a}	0.5405^{a}	0.0485^{a}	0.5931^{a}	0.8628^{a}	0.8847^{a}	-0.5827	-0.5827	-0.4914	0.2191	0.8958	-0.1474
**NO assay**	-0.4809^{b}	-0.3338^{b}	-0.2068^{b}	-0.5810^{b}	0.5789^{b}	0.0960^{b}	0.5127^{b}	-0.2281^{b}	-0.1743^{b}	0.6242	-0.3447	0.5762	0.2370	-0.2364	0.4267
**Reducing power**	-0.6917^{c}	0.7784^{c}	0.4117^{c}	-0.5560^{c}	0.7735^{c}	0.0282^{c}	0.7879^{c}	0.5054^{c}	0.5659^{c}	-0.5652	-0.0586	-0.5259	0.3773	0.5389	0.3352
**Iron chelation**	0.4752^{d}	-0.7511^{d}	-0.5845^{d}	0.3237^{d}	-0.4933^{d}	0.1177^{d}	-0.5857^{d}	-0.3881^{d}	-0.5101^{d}	0.8073	-0.1513	0.8228	-0.4568	-0.4579	-0.1194
**LP inhibition**	-0.5426^{e}	0.1178^{e}	0.2503^{e}	-0.4861^{e}	0.7426^{e}	0.2304^{e}	0.7445^{e}	-0.1381^{e}	-0.1105^{e}	0.0733	-0.0818	0.0138	0.2030	-0.1304	0.5505
**AChE inhibition**	-0.8019^{f}	0.1896^{f}	0.1745^{f}	-0.8011^{f}	0.8168^{f}	-0.0210^{f}	0.8727^{f}	0.0126^{f}	0.2374^{f}	-0.1043	-0.2877	-0.2167	0.7050	0.0960	0.2916
**α-Glu inhibition**	-0.2135^{b}	0.0276^{e}	0.7934^{a}	-0.1238^{b}	0.2623^{f}	-0.4323^{g}	0.4453^{f}	-0.2962^{f}	-0.1007^{d}	-0.2262	0.5093	-0.3889	0.5791	-0.2103	0.1244

Values displaying different letters within each column are significantly different according to the Tukey's multiple comparisons test at 95% confidence level. Coefficient values from 0.10 to 0.39 indicate weak correlation. values from 0.40 to 0.69 indicate moderate correlation. values from 0.70 to 0.89 indicate strong correlation and values from 0.90 to 1.00 indicate very strong correlation. Abbreviations: A – Apigenin, A-7-G - Apigenin-7-glucoside, AChE – Acetylcholinesterase, α-Glu - α-Glucosidase, CA – Caffeic acid, ChA - Chlorogenic acid, FA - Ferulic acid, L- Luteolin, L-7-G – Luteolin-7-glucoside, LP - Lipid peroxidation, pCA - *p*-Coumaric acid, Q – Quercetin, R – Rutin, RA - Rosmarinic acid, TA - Total anthocyanins, TF - Total flavonoids, TPA - Total phenolic acids, TT – Total tannins.
